# Transfer and Decontamination of *S*. *aureus* in Transmission Routes Regarding Hands and Contact Surfaces

**DOI:** 10.1371/journal.pone.0156390

**Published:** 2016-06-09

**Authors:** Pernilla Arinder, Pär Johannesson, Ingela Karlsson, Elisabeth Borch

**Affiliations:** 1 Food and Bioscience, SP Technical Research Institute of Sweden, Lund, Sweden; 2 Mechanics Research, SP Technical Research Institute of Sweden, Borås, Sweden; 3 Food and Bioscience, SP Technical Research Institute of Sweden, Göteborg, Sweden; Agricultural University of Athens, GREECE

## Abstract

Hand hygiene, cleaning and disinfection are pre-requirements for hygiene management in hospital settings and the food industry. In order to facilitate risk management, different contamination scenarios and interventions need to be evaluated. In the present study data on transfer rates and reductions of *Staphylococcus aureus* were provided in an experimental set-up using artificial skin. Using this methodology, test persons were not exposed with pathogenic bacteria. An exposure assessment model was developed and applied to evaluate different contamination routes and hygiene interventions. The transfer rates of *S*. *aureus* from inoculated VITRO-SKIN^®^ to fomites were calculated from blotting series. The VITRO-SKIN^®^ was more prone to spread bacteria than fomites. When different surfaces were cleaned, the reduction of *S*. *aureus* varied between <1 and 7 log CFU. It could not be concluded that a certain coupon material, cleaning agent, cleaning wipe, soiling or humidity consistently resulted in a high or low reduction of *S*. *aureus*. The reduction of *S*. *aureus* and *E*. *coli* during hand washing was evaluated on artificial skin, VITRO-SKIN^®^. The reduction of *E*. *coli* on VITRO-SKIN^®^ was similar to the log reduction obtained when washing human hands. The *S*. *aureus* count on a human hand was both calculated in different scenarios describing different contamination routes starting from a contaminated hand using the exposure assessment model, and measured on an experimental setup using VITRO-SKIN^®^ for validation. A linear relationship was obtained between the analysed level of *S*. *aureus* and the calculated level. However, the calculated levels of *S*. *aureus* on the VITRO-SKIN^®^ in the scenarios were 1–1.5 log lower than the analysed level. One of the scenarios was used to study the effect of interventions like hand washing and cleaning of surfaces.

## Introduction

Health Care Associated Infections (HCAI) are a major problem. Approximately 5 million cases of HCAI occur annually in Europe [1]. This results in 25 million extra patient days in hospitals, at a cost of €13–24 billion [1]. Methicillin resistant *Staphylococcus aureus* (MRSA) is one of the organisms known for causing Health Care Associated Infections. Other microorganisms, where the hospital environment is important, are vancomycin resistant *Enterococcus* spp. (VRE), *Clostridium difficile*, *Acinetobacter* spp. and norovirus [2]. Contaminated surfaces contribute to the transmission of pathogenic bacteria in hospitals [3]. Contact surfaces in hospitals may have 100−10^5^ CFU/cm^2^ of MRSA or Methicillin sensitive *Staphylococcus aureus* MSSA [4]. Fomites and hands play a significant role in the spread of microorganisms, not only in hospitals but also in nursing homes, child care settings, the food industry and in restaurants [5]. Hand hygiene, cleaning and disinfection are pre-requirements for hygiene management in hospital settings and the food industry to minimise the risk of Health Care Associated Infections and foodborne infections [6] [7] [8].

In order to facilitate risk management, different contamination scenarios and interventions need to be evaluated and compared. Mathematical models illustrating the transfer and reduction of microorganisms are used in quantitative microbial risk assessments (QMRA; [9]). Both laboratory and field investigation data are useful in the models [10]. Several exposure assessment models, describing cross contamination and contamination routes, have been published. Different scenarios were performed to assess *Listeria monocytogenes* cross contamination during the handling of cooked ham, using models to ascertain how they affected the food safety objectives [11]). In a QMRA performed by Stals et al. 2015 [12], the transmission of norovirus was demonstrated, based on observations of handling during preparation of sandwiches and literature data on transfer rates. Risk assessment of hand washing efficacy showed that the primary factors affecting the final bacterial count on the hands were the sanitizer, soap and drying method [13]. The effect of different antimicrobial hand products during handling of ground beef was assessed using an exposure assessment model used for probability simulation [9]. A model for transmission of gastrointestinal viruses was developed by Mokhtari and Jaykus 2009 [14] in order to compare different mitigations.

Data on transfer rates are necessary for the performance of predictions and simulations of contamination routes. The reduction of the specific bacteria needs to be quantified in order to predict the effect of interventions, like washing and disinfection, on the contamination scenarios. Although literature data exists for transfer rates and reductions for some microorganisms, further knowledge is required to enable the simulation of scenarios. Transfer rates are reported for methicillin-resistant *S*. *aureus* between pig skin and different fomites [15]. Transfer rates of *S*. *aureus* have also been evaluated between finger tips and bed rails of different materials showing differences between different surface materials and in the presence of organic soiling [16]. The effect of different cleaning cloths and cleaning agents on different surface materials has been evaluated in studies showing a variation in bacterial reduction [16] [17] [18].

One difficulty when it comes to provide data on *S*. *aureus* are the safety concerns for test persons, who participate in handwashing studies or in studies to determine transfer rates between fingers and fomites. This restricts the amount of available data. The use of alternative test methods, such as artificial skin, would give increased opportunities to conduct studies of *S*. *aureus*.

The aim of this study was to produce data on transfer rates of *S*. *aureus* between VITRO-SKIN^®^ and different surface materials, and to determine the reduction of *S*. *aureus* during cleaning of surfaces and washing of VITRO-SKIN^®^.The transfer rate of *E*. *coli* was determined between VITRO-SKIN^®^ or human skin and stainless steel as a reference. The reduction of *E*. *coli* during washing of VITRO-SKIN^®^ was compared with the one on human skin. An exposure assessment model for *S*. *aureus* was developed and applied for different contamination routes and hygiene interventions.

## Material & Methods

Voluntary persons participated in a handwashing study performed in accordance with the methodology described in the methodology in SS EN 1500. The ethic committee (EPN Gothenburg Dnr: 300–15) concluded that the study was not included in the type of research that covered by the ethic review act.

### Bacterial Strains

The strains of *Staphylococcus aureus* used in the experiments were a selection of isolates from human and foods (ATCC 25923, CCUG 1800T, CCUG 25924, ATCC1969, CCUG 35611, SIK 691 and SIK 692). For experiments with *Escherichia coli*, *E*. *coli* K12 (NTCC 10538) was used. This strain is chosen in the standard test method SS-EN 1500 for evaluation of hygienic hand rub.

### Determination of Bacterial Count

The numbers of *S*. *aureus* and *E*. *coli* were analysed by serial dilution in Peptone Water (Bacto^™^ Peptone Becton, Dickinson and Company Sparks MV 2152, USA/38800 Le Pont de Claix, France) and subsequent plate count on TSA (Trypton Soy Agar, Oxoid LTD, Basingstoke, Hampshire, UK) incubated for 24 hours at 37°C.

### Preparation of Artificial Skin

VITRO-SKIN^®^N-19 (IMS Inc) was used to simulate human skin during the evaluation of transfer rate and bacterial reduction during hand washing. Pieces of VITRO-SKIN N-19 were cut into 5x5 cm squares and placed in a chamber with 15% glycerine for 16–24 hours in order to achieve humidity. When the chambers were opened, the humidity inside was 75–95% RH. The humidity was monitored using Tinyview 4500 (Tinytag). The experiments were performed in a climate room with 50–57% RH in order to keep the skin humid.

### Preparation of Fomites of Different Materials

For the evaluation of transfer rates, 5x5 cm fomites were prepared from stainless steel (ss2346), cutting board (HDPE, Daloplast, item no. 360D-00U), melamine laminate (Fyndig IKEA, item no. 502.375.33 high-pressure laminate melamine, foil), tile (K5056068 Ral 9016), milk carton, plastic apron (Selefa) and cotton fabric (laboratory coat). The fomites were treated before the tests to minimise the background flora. The stainless steel fomites were machine washed with Neodisher LaboClean FT, using sodium hypochlorite (1–5%), potassium hydroxid (5–15%), and subsequently dry sterilised at 180°C (Termaks) for three hours. The HDPE (cutting board), melamine laminate and tile were hand washed with YES/Fairy (Procter & Gamble) and subsequently treated with 70% ethanol. The milk carton was sprayed with 70% ethanol. The plastic apron was used directly after removal from the package. The cotton textile (laboratory coat) had been newly washed at a hospital laundry.

To evaluate the cleaning effect, the stainless steel, tile and melamine laminate test surfaces (5x10 cm) were cleaned as described above.

### Preparation of Inoculum

The bacterial solutions of *S*. *aureus* were prepared from separate pre-cultures in nutrient broth (NB, Oxoid LTD, Basingstoke, Hampshire, UK; 37°C, 20 hours) of the seven strains. 0.15 ml of each strain culture was mixed into 9 ml of Difco^™^ D/E Neutralizing Broth (Becton, Dickinson and Company Sparks MD 21152, USA, 38800 Le Pont de Claix, France) to produce a *S*. *aureus* 7-strain cocktail containing about 6 log CFU/ml.

The solution of the *E*. *coli* strain was prepared from two tubes of subcultures each containing 5 ml of Tryptone Soy Broth (TSB, Oxoid LTD, Basingstoke, Hampshire, UK) cultured at 37°C for 24 hours. The tubes were transferred to two bottles, each containing 1 litre of TSB, for further culturing at 37°C for 24 hours.

### Evaluation of *S*. *aureus* Transfer between Fomites and Artificial Skin

#### Transfer of *S*. *aureus* from inoculated VITRO-SKIN N-19 to fomite

To evaluate the transfer rate of *S*. *aureus* from VITRO-SKIN N-19 to different fomites, two pieces of VITRO-SKIN N-19 were each inoculated with 0.2 ml of the *S*. *aureus* solution. The inoculum was spread over the skin with a spreader for one minute. One of the skin pieces was transferred to a stomacher bag with 10 ml Peptone Water (Bacto ^™^ Peptone Becton, Dickinson and Company Sparks MV 2152, USA/38800 Le Pont de Claix, France) for determination of the inoculated *S*. *aureus* concentration. The other piece was placed on the bottom of a petri dish.

In order to evaluate the transfer rate, 21 fomites of a certain material were pressed in blotting series onto the inoculated VITRO-SKIN N-19. During each series, bacterial counts were determined for eight subsequent blotting. The blotting series was repeated three times for each fomite. Each estimated transfer rate was based on 24 measurements. A pressure of 0.5 kg for 10 seconds was chosen to mimic the pressure assessed when holding a coffee cup or using a door handle. The blotting procedure was performed on a balance (Mettler PC 4400, Mettler Toledo) to obtain the 0.5 kg pressure. The bacterial count on the fomite numbers 1, 3, 6, 9, 12, 15, 18 and 21 in the blotting order were sampled using a cotton swab (cotton-tipped applicator, ONEMED Group Oy, Finland). The swab was placed in 3 ml Peptone Water (Bacto ^™^ Peptone Becton, Dickinson and Company Sparks MV 2152, USA/38800 Le Pont de Claix, France) and vortexed, and the bacterial concentration in the solution was determined.

#### Transfer from inoculated fomites of different materials to VITRO-SKIN N-19

To evaluate the transfer rate of *S*. *aureus* from different fomites to VITRO-SKIN N-19, two coupons were each inoculated with 0.1 ml of *S*.*aureus* solution. The coupons were dried for 15–20 minutes in a laminar flow cabinet (Holten LaminAir, Heto Holten A/S, Denmark). One of the coupons was sampled, using one moisturised swab and one dry swab to determine the initial concentration of *S*. *aureus*. The swab was moisturised by dipping in Peptone Water (Bacto^™^ Peptone Becton, Dickinson and Company Sparks MV 2152, USA/38800 Le Pont de Claix, France).

The other fomite was pressed at 0.5 kg for 10 seconds in a blotting series onto 21 pieces of VITRO-SKIN N-19 placed in petri dishes. Seven pieces of VITRO-SKIN N-19 for the blotting series were taken out of the humidity chamber one at a time, in order to maintain the humidity of the skin. After the blotting procedure, the VITRO-SKIN^®^ N-19 numbers 1, 3, 6, 9, 12, 15, 18 and 21 in the blotting order, were placed in stomacher bags with 10ml of Peptone Water. The bacterial count in the Peptone Water was determined. The blotting series was repeated three times.

### Calculation of Transfer Rate

The bacterial transfer rate was calculated from the results of the blotting series. The transfer rate from inoculated VITRO-SKIN^®^ N-19 (donor surface) to a fomite (recipient surface) was calculated using the bacterial counts on coupons versus blotting order. The transfer rate from inoculated fomite (donor surface) to VITRO-SKIN^®^ N-19 (recipient surface) was calculated using the bacterial counts analysed on VITRO-SKIN^®^ N-19 pieces versus blotting order. Bacterial count remaining on the donor surface after *k* blottings is modelled by:

$$N_{k}=N_{0}\cdot R^{k}$$ (1)

where *N*_0_ is the initial count on the donor surface and *R* is the proportion (after each blotting) that is left at the surface, and is used for describing the reduction.

The bacterial count on the recipient surface after *k* blottings is thus modelled by:

$$Y_{k}=N_{k-1}\cdot \left(1-R\right)=N_{0}\cdot R^{k-1}\cdot \left(1-R\right)$$ (2)

where 1−*R* is the proportion that transfers from the donor surface to the recipient surface.

In logarithmic scale the model of the bacterial count on skin after *k* blottings is:

$${\text{log}}_{10}Y_{k}={\text{log}}_{10}N_{0}+\left(k-1\right){\text{log}}_{10}R\;+{\text{log}}_{10}\left(1-R\right)={\text{log}}_{10}N_{0}+{\text{log}}_{10}\frac{\left(1-R\right)}{R}+k\cdot {\text{log}}_{10}R$$ (3)

This forms a linear regression model of the logarithmic bacterial count, log_10_ *Y*_*k*_, versus the number of blottings, *k*, where the slope of the regression line is the logarithm of the transfer rate, denoted by b = log_10_ *R*. Thus, the transfer rate in % (the proportion that is transferred from the donor surface to the recipient surface) is calculated as *T* = (1 − *R*) ⋅ 100 = (1 − 10^*b*^) ⋅ 100.

### Evaluation of Cleaning of Contaminated Fomites

To evaluate the cleaning effect, cleaned stainless steel, tile and melamine laminate test surfaces (5x10 cm) were covered with 0.2 ml of the soiling-solution and subsequently dried in a laminar flow cabinet (Holten LaminAir, Heto Holten A/S Denmark) for 15–20 minutes until the surface appeared dry. Two types of soiling solutions were used; (i) a mix of Peptone Water (5 ml), *S*. *aureus* inoculum (4.9 ml) and Bovine Albumin (Fluka, 0.1 ml *3 g/l), or (ii) mix of Peptone Water (5 ml), *S*. *aureus* inoculum (4.9 ml) and egg-milk mix (1 whole egg + 1 dl milk (3% fat); (0.1 ml)).

Two different wipes were used for the cleaning; “Blue” made of viscose and polyester (EasiTex torkduk 47, Gunnar Engstrand AB) and “White” made of “nyfiber” (Tvättlapp tissue item no. 71300, Abena AB). Before cleaning, the wipes of 8.5–8.7 g were moistened with 5 g and 17 g of the different cleaning and disinfection solutions to get low and high moistened wipes, respectively. The moistened wipes were kept in stomacher bags for 1 hour before use in order to even out the moisture. The cleaning/disinfectant used was Allrengöring Sprint 200 free (1% solution, Diversey Sverige AB), Rely + On Virkon (1% solution, Dupont) and Dax 70+ surface disinfection (Opus Health Care AB).

The moistened cleaning wipes were folded three times and attached to a “cloth holder”. The “cloth holder” had a contact surface of 50cm^2^ and a weight of 500g. The cloth holder with a cleaning wipe was wiped slowly over the soiled surface twice with a 90° turn in between. A fresh, clean part of the cloth was used for each wipe. 10 minutes after wiping, the surface was sampled using three cotton swabs, two moistened and one dry. The swabs were placed in 3 ml of Difco^™^ D/E Neutralizing Broth (Becton, Dickinson and Company, Sparks MD 21152, USA/38800 Le Pont de Claix, France) and wortexed (Vibrofix VF1 Electronic, Janke & Kunkel, IKA Labortechnik). The bacterial count in the solution was analysed. The tests were performed in three replicates.

An Analysis of Variance (ANOVA) was performed in order to investigate the influence of different factors on the reduction, see e.g. [19].

### Reduction of *E*. *coli* during Hand Washing

#### Hand washing

The methodology in SS EN 1500 “Chemical disinfectants and antiseptics -Hygienic hand rub—Test method and requirements (phase 2)”, where *E*. *coli* is the test organism, was used to evaluate the hand- washing effect. Twelve volunteers prepared their hands by washing them with soap and drying them on paper towels. To contaminate their hands, the test persons dipped them into a two–litre solution of *E*. *coli* (8.6 log CFU/ml) for five seconds. Their hands were subsequently air-dried by holding them horizontally, with the fingers spread, and slowly rotating them back and forth for three minutes. To determine the initial concentration of *E*. *coli*, the fingertips of one hand were rubbed for one minute onto the base of a Petri dish containing 10 ml of TSB, and the bacterial count in the TSB was analysed.

Two washing strategies were evaluated. The test persons washed their hands either with soap solutions (Dax, mild tvål, Opus Health Care AB) or with a soap solution followed by hand drying with paper towels and hand disinfection (Dax, Handdesinfektion 70+ Opus Health Care AB). The remaining *E*. *coli* concentration on the hand/fingers after washing was analysed on the second hand in the same way as the initial concentration, but with neutralisation solution according to the standard SS EN 1500 (Tween 80, saponin, histidine, cysteine) in the TSB solution according to the methodology in SS EN 1500. 3 ml of soap solution was placed on the hands, which were washed according to a washing scheme. The fingers were then rinsed with water for five seconds or disinfected with 3 ml of 70% hand disinfection.

#### Reduction of *E*. *coli* and S. aureus during washing of artificial skin

A piece of VITRO-SKIN N-19 was inoculated with 0.2 ml of *E*. *coli* or *S*. *aureus* solution spread over the skin using a spreader for one minute and was subsequently dried for one minute. The same type of soap solution used for cleaning hands was applied on the skin. A spreader covered with another piece of VITRO-SKIN N-19 was used for rubbing the inoculated skin to simulate hand washing. The skin was rubbed 20 times while rotating the skin in 90° angels to achieve the same amount of foam as normal hand washing. After rubbing, the skin was rinsed in water by dipping the skin in three bottles of tap water. The skin was transferred to 10 ml of neutralisation solution, according to the SS EN 1500 standard (TSB with Tween 80, saponin, histidine and cysteine). After homogenising, the bacterial concentration in the solution was determined.

### Transfer of *E*. *coli* between Stainless Steel and Thumbs

Both the thumbs of ten test persons were contaminated with 0.025 ml of *E*. *coli* K12 solution. The inoculum was spread on the thumbs with a pipette tip. The inoculum was then air-dried for ~6 minutes until dry. One of the thumbs was pressed onto a stainless steel coupon placed on a balance (Mettler PC 4400, Mettler Toledo) at a pressure of 0.5 kg for 10 seconds. To evaluate the transfer from stainless steel to the thumbs, stainless steel coupons were inoculated with 0.1 ml of *E*. *coli* solution and the inoculum was air-dried for 12 minutes. One of the thumbs of the test persons was pressed onto a stainless steel coupon at a pressure of 0.5 kg for 10 seconds. The bacterial count was determined on the thumb and on the stainless steel coupons. The thumbs were rubbed for 1 minute on the base of a Petri dish containing 10 ml of TSB, according to the methodology described in SSEN 1500. The TSB solution was then diluted and spread on TSA plates. The coupon was sampled using 2 cotton swabs, one moistened in Peptone water and one dry. The swabs were placed in 3 ml of neutralisation solution (Tween 80, saponin, histidine, cysteine) before the concentration of *S*. *aureus* in the solution was analysed.

### Transfer of *S*. *aureus* in Different Scenarios

The transfer of *S*. *aureus* was evaluated at different stages in three scenarios, describing contacts between hand and stainless steel coupon and vice versa.

1. Hand 1 touches a stainless steel door handle. The door handle is touched by Hand 2 and subsequently by Hand 3.
2. Hand 1 touches a door handle that is subsequently touched by Hand 2. Hand 2 touches a laminate bench top. Hand 3 touches the same spot on the bench.
3. Hand 1 touches a stainless steel door handle. Hand 2 touches the same door handle and then touches a laminate bench top. Hand 3 touches the bench top touched by Hand 2 and then touches the door handle. Hand 4 touches the door handle that has been touched by Hand 1 and Hand 3.

The starting point of the scenarios was always a VITRO-SKIN^®^ inoculated with 0.2 ml of bacterial solution. The bacterial counts on the coupons and the VITRO-SKIN^®^ were analysed as described above.

### Transfer and Reduction Modelling in a Contamination Scenario

The bacterial count on hands and fomites was modelled for transfer of bacteria and reduction of bacteria at different stages in a scenario, using a developed Transfer Reduction Model (TRM). Propagation of the variances in the bacterial count through the chain of events/actions was calculated using VMEA (Variation Mode and Effect Analysis; [20] [21]). The bacterial count and variance were calculated at each stage of the contamination chain.

The bacterial count on surfaces in the scenarios after *n* transfers was modelled according to:

$$N=N_{0}\cdot T_{1}\cdot T_{2}\cdot \ldots \cdot T_{n}=N_{0}\cdot \prod_{k=1}^{n}T_{k}$$ (4)

where *N* is the predicted bacterial count (CFU), *N*_0_ the initial count (CFU), and *T*_*k*_ the transfer or reduction proportion (value between 0 and 1).

By taking the logarithm of model (4), a linear relation is obtained for the transfer reduction model (TR Model):

$${\text{log}}_{10}N={\text{log}}_{10}N_{0}+\sum_{k=1}^{n}{\text{log}}_{10}T_{k}$$ (5)

The mean and variance for the bacterial count (log_10_ CFU) are:

$$m=\text{E}\left[{\text{log}}_{10}N\right]=\text{E}\left[{\text{log}}_{10}N_{0}\right]+\sum_{k=1}^{n}\text{E}\left[{\text{log}}_{10}T_{k}\right]$$ (6)

$$s^{2}=\text{Var}\left[{\text{log}}_{10}N\right]=\text{Var}\left[{\text{log}}_{10}N_{0}\right]+\sum_{k=1}^{n}\text{Var}\left[{\text{log}}_{10}T_{k}\right]$$ (7)

Using the approximation that the bacterial count given as log CFU is normally distributed, we will get a 95% prediction interval for the bacterial count according to:

m±2.0⋅s (8)

Calculations were performed in Excel where the scenarios were built. The input values of initial count, transfer rates and reductions were given in log_10_ scale. Transfer both from and to the fomites and skins were taken into account.

### Statistical Evaluation

The TR Model was implemented in Excel as described above. Analyses of variance were performed using the statistical software R (version 3.2.0), which is available as Free Software (http://www.r-project.org/). A linear model was used where independent and normally distributed errors were assumed. The functions used in R were boxplot (boxplots with notches), aov (Analysis of Variance), TukeyHSD (Tukey’s Honest Significant Difference), and pairwise.t.test (Pairwise t-tests).

## Results

### Transfer of *S*. *aureus* between Fomites and VITRO-SKIN^®^

Transfer rates of *S*. *aureus* were determined by repeated blotting from artificial skin VITRO-SKIN^®^ to fomites, and vice versa from fomites to VITRO-SKIN^®^.

**Fig 1:**
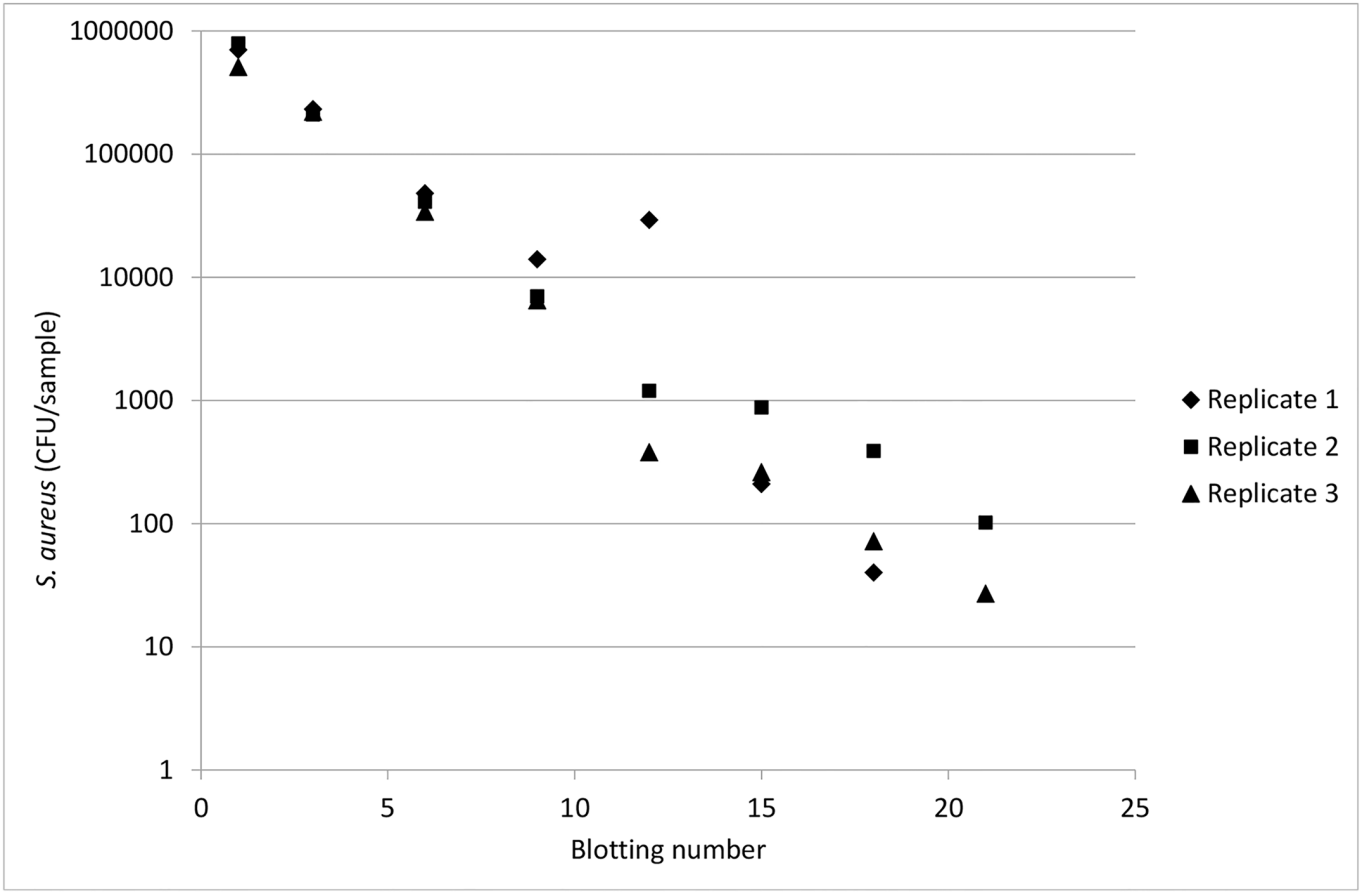
The number of *S*. *aureus* analysed on stainless steel after different numbers of blotting to an inoculated piece of VITRO-SKIN^®^.

The relationship between the logarithmic number of bacteria on the fomite and the order in the blotting series was linear for all fomites, as exemplified by stainless steel in Fig 1.

The estimated transfer rates of *S*. *aureus* from inoculated VITRO-SKIN^®^ to fomites are shown in Table 1. Each estimate of the transfer rate is the mean of three replicates, where each replicate is calculated from a blotting series of 21 blottings, every third of these were used for bacterial counts determination. This means that each estimated transfer rate is based on 24 measurements; see Fig 1 for an example. The results in Table 1 are derived from the statistical analysis of the logarithmic values, see Eq (3). However, for ease of presentation and interpretation of the results, the logarithmic values are not presented, but instead the estimated transfer rates in % are presented together with their corresponding standard errors. The standard error is the standard deviation of the estimated mean transfer rate, and has been calculated by approximation from the logarithmic values. The mean transfer rate (n = 3 blotting series) from inoculated VITRO-SKIN^®^ to stainless steel, cutting board, milk carton, plastic apron, laminate and tile was 33.1%-39.2% with a standard deviation of the estimated mean value between 0.3% and 2.0% (Table 1). The transfer rate from inoculated VITRO-SKIN^®^ to textile was lower, 6.7% with a high standard deviation of ±16.5%. The highest transfer rate was from contaminated VITRO-SKIN^®^ to stainless steel, where the estimated transfer rate was 39.2%±1.9%. The fomites studied received a similar contamination from the infected VITRO-SKIN^®^, except for the cotton fabric.

**Table 1:** Calculated transfer rate of *S*. *aureus* between VITRO-SKIN^®^ and coupons of different materials.

	Transfer rate (%)*
Transfer of *S*. *aureus* from contaminated VITRO-SKIN^®^ to:	
• Stainless steel	39.2 ± 1.9
• Cutting board (HDPE)	33.1 ±1.5
• Cardboard (milk carton)	34.5 ± 0.9
• Textile (cotton fabric from laboratory coat)	6.7 ± 16.1
• Plastic apron	33.9 ± 2.0
• Melamine laminate	38.2 ± 0.2
• Tile	34.1 ± 1.3
Transfer of *S*. *aureus* to skin from:	
• Stainless steel	5.3 ± 1.1
• Cutting board (HDPE)	4.0 ± 1.1
• Cardboard (milk carton)	7.4 ± 2.7
• Textile	Not evaluated
• Plastic apron	Not evaluated
• Melamine laminate	1.5 ± 0.7
• Tile	9.4 ± 2.7

* Calculated from blotting series (n = 3) where 21 blottings were performed.

The transfer rates of *S*. *aureus* from inoculated VITRO-SKIN^®^ to fomites were significantly higher than from inoculated fomites to VITRO-SKIN^®^ (Table 1). The VITRO-SKIN^®^ was, thus, more prone to spread bacteria than a fomite. The average transfer rates of *S*. *aureus* from inoculated fomites to VITRO-SKIN^®^ varied between 1.5% and 9.4%, and the standard deviation of the estimated mean value varied between 0.7% and 2.7% (Table 1). The highest transfer rate from inoculated surfaces to VITRO-SKIN^®^ (9.4%±2.7%) was found for tile. The lowest transfer rate was from contaminated laminate to VITRO-SKIN^®^ (1.5%±0.7%).

**Fig 2:**
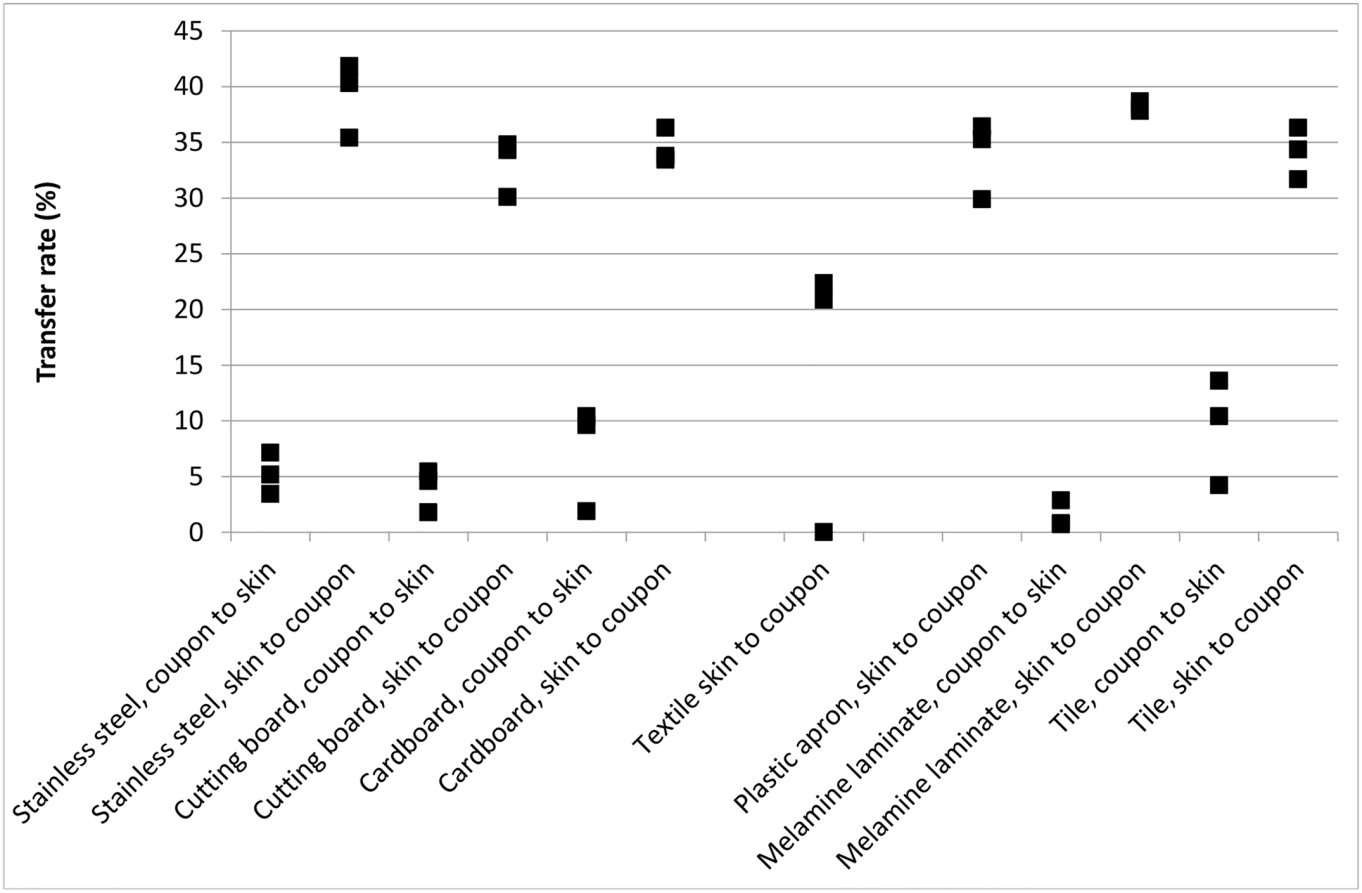
Transfer rate for transfer of *S*. *aureus* between VITRO-SKIN^®^ and stainless steel, cutting board, milk carton, melamine laminate and tile coupons.

There was no significant difference in the transfer rates between the inoculated VITRO SKIN^®^ and the different fomites; irrespective of recipient and donor (Fig 2).

### Transfer Rate of *E*. *coli* between Stainless Steel and VITRO-SKIN^®^ or Human Skin

A comparison was made between the transfer rates determined using VITRO-SKIN^®^ or human skin (thumbs). *E*. *coli* was used as a test organism instead of *S*. *aureus*.

The transfer rate of *E*. *coli* from inoculated VITRO-SKIN^®^ to stainless steel was 45.6%±3.6% and 4.1%±0.4% from contaminated stainless steel to VITRO-SKIN^®^ (n = 3 blotting series). The transfer rates were of the same magnitude as for *S*. *aureus*, 39.2%±1.9% and 5.3%± 1.1% (Table 1).

The initial number of *E*. *coli* on thumbs before touching the stainless steel coupons was 5.0±1.4 log CFU/sample and after touching the stainless steel 4.4±1.3 log CFU/sample (n = 10). This resulted in a transfer rate of 75% for *E*. *coli* from finger tips to stainless steel, calculated on the arithmetic values. This was higher than the transfer rate using VITRO-SKIN^®^.

The initial number of *E*. *coli* on inoculated stainless steel coupons was 5.7±0.2 log CFU/sample. After touching with thumbs, the level of *E*. *coli* on the coupons was 4.9±0.6 log CFU/sample (n = 9). This resulted in a transfer rate of 85% for *E*. *coli* from stainless steel to finger tips. This was higher than the transfer rate using VITRO-SKIN^®^.

In conclusion, when using a traditional experimental set-up with human fingers or hands, the transfer rates were higher than when using VITRO-SKIN^®^ and blotting series under carefully-controlled conditions, with regard to humidity, temperature, pressure and pressure time.

### Reduction of *S*. *aureus* on Fomites during Cleaning Using Wipes

Two types of cleaning fabrics, three cleaning agents and two humidity levels were used for the cleaning of coupons inoculated with *S*. *aureus* in two types of soiling (bovine albumin or egg). The reduction of *S*. *aureus* during cleaning varied between <1 and >7 log CFU (Table 2). In 27 of the 72 combinations of cleaning factors, the mean logarithmic reduction of *S*. *aureus* was higher than 5.0 log CFU. It could not be concluded that a certain coupon material, cleaning agent, cleaning wipe, soiling or humidity consistently resulted in a high or low reduction of *S*. *aureus*. An example of combinations resulting in a high reduction of *S*. *aureus* was cleaning of a tile soiled with Bovine Alumin, using Dax 70 as the cleaning agent. Less reduction was achieved when the tile was soiled with egg.

**Table 2:** Reduction of *S*. *aureus* on different surface materials inoculated with two types of soiling using two types of cleaning wipes, three cleaning agents and two humidity levels (n = 3).

Cleaning agent and cleaning wipe	Reduction of *S*. *aureus* (log CFU/surface)					
	Stainless steel + BA soiling	Stainless steel + Egg soiling	Laminate + BA soiling	Laminate + egg soiling	Tile + BA soiling	Tile + egg soiling
Allrengöring						
Blue, high humidity	>6.2	5.7±0.2	4.0±0.1	3.5±0.4	6.3±0.1	5.9±1.4
Blue, low humidity	5.2±1.9	4.2±3.1	3.5±0.7	1.0±0.8	6.0±0.3	1.9±2.7
White, high humidity	5.2±0.2	>6.7	3.5±0.3	3.1±0.7	4.5±1.3	5.0±0.4
White, low humidity	3.3±0.1	5.5±0.7	1.1±0.2	0.5±0.2	3.5±0.2	2.0±1.5
Virkon						
Blue, high humidity	2.2±0.5	6.7±0.0	5.3±0.3	3.6±0.4	5.2±0.6	5.1±1.0
Blue, low humidity	2.2±0.4	2.0±1.4	5.0±1.6	2.1±0.2	5.3±0.5	1.5±2.0
White, high humidity	3.1	6.1±0.3	4.9±1.0	5.4±0.3	5.9±0.3	4.6±1.2
White, low humidity	2.6±1.1	4.8±0.6	3.2±0.4	0.5±0.4	3.8±1.9	2.1±1.3
Dax 70						
Blue, high humidity	2.8±0.8	6.6±1.0	4.3±1.0	7.1±1.0	6.1±1.1	2.1±1.0
Blue, low humidity	5.2±1.1	0.3±0.3	2.5±0.4	7.1±0.3	6.1±1.9	0.2±0.2
White, high humidity	3.4±1.0	4.1±1.0	4.7±1.0	1.4±1.0	6.6±1.0	4.5±1.0
White, low humidity	4.7±1.1	0.7±0.3	1.3±0.7	0.5±0.2	6.6±1.0	1.3±0.5

**Fig 3:**
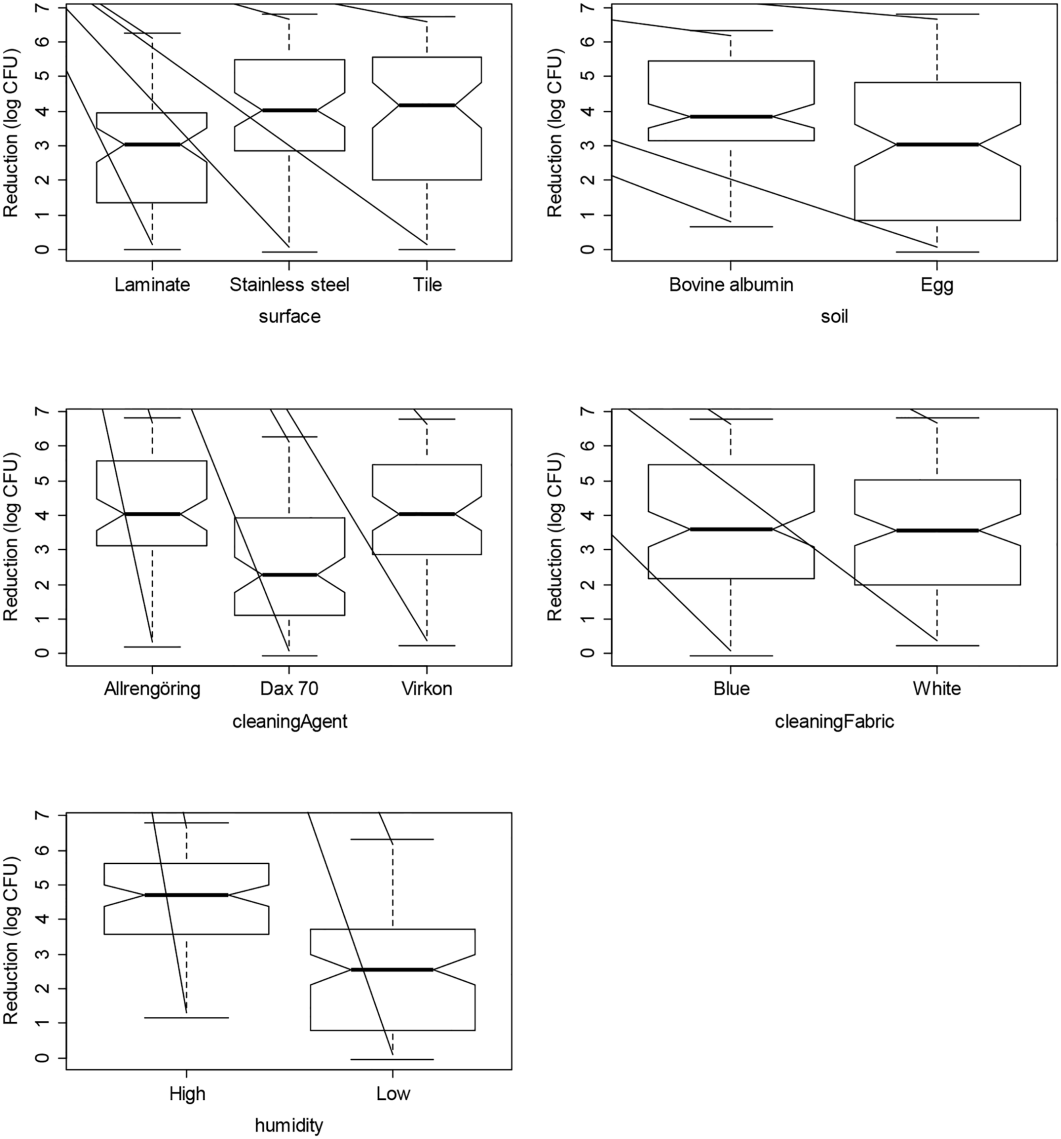
Boxplots with notches to indicate significant differences, illustrating the reduction of *S*. *aureus* for the parameters; material, soiling, cleaning agent, cleaning cloth and humidity.

In Table 3 the ANOVA analysis shows that most factors (coupon material, soiling, cleaning agent and humidity) were statistically significant for the reduction of *S*. *aureus*. The only exception was the type of cleaning wipe. The laminate was the most difficult material to clean (p<0.001). There was no significant difference in the cleaning effect on stainless steel and tile. However, the reduction of *S*. *aureus* was significantly higher using a humid cloth than a less humid (p<0.001). The boxplots in Fig 3 give a graphical illustration of the data, where the notches give an indication of significant differences. If the notches of two plots do not overlap this is evidence that the two medians differ [22].

**Table 3:** Analysis of Variance Table for reduction (log CFU) of *S*. *aureus* on different surface materials inoculated with two types of soiling using two types of cleaning wipes, three cleaning agents and two humidity levels.

Factor	Sum of Squares	df	Mean Squares	F-value	p-value
Coupon material	58.06	2	29.03	16.50	2.23E-07
Soiling	56.65	1	56.65	32.20	4.63E-08
Cleaning wipe	1.30	1	1.30	0.74	0.3904
Cleaning agent	99.01	2	49.50	28.14	1.53E-11
Humidity	197.95	1	197.95	112.51	< 2.2e-16
Residuals	365.98	208	1.76		

The results demonstrate the necessity to have good knowledge of various combinations of cleaning factors in order to choose the most efficient cleaning combination.

### Washing of Hands Contaminated with *E*. *coli*

Hand washing with soap reduced the number of *E*. *coli* on the contaminated fingertips from 5.7 ±0.5 log CFU/sample to 2.8±0.5 log CFU/sample. Hand washing with soap and subsequent disinfection reduced the level of *E*. *coli* from 5.9±0.5 log CFU/sample to 1.8 ±1.0 log CFU/sample. Soap plus disinfection resulted in 1.2 log greater reduction than only soap.

### Washing of VITRO-SKIN^®^ Contaminated with *E*. *coli* or *S*. *aureus*

The reduction of *S*. *aureus* and *E*. *coli* was evaluated on artificial skin, VITRO-SKIN^®^. The washing procedure was designed to simulate hand washing according to SS EN 1500 using soap, rubbing and rinsing. The initial count on the inoculated VITRO-SKIN^®^ was 7.6±0.1 log *S*. *aureus*/sample and 7.7±0.1 log *E*. *coli*/sample. After rinsing the VITRO-SKIN^®^ with only water, *S*. *aureus* was reduced by 0.5±0.1 log CFU/sample and *E*. *coli* by 1.6± 0.4 log CFU/sample. The bacterial reduction using soap solution and subsequent water rinse was 2.0 ± 0.4 log CFU/sample for *S*. *aureus* and 2.5± 0.5 log CFU/sample for *E*. *coli*. Soap plus water rinse resulted in a 1.5 log reduction and a 0.9 log greater reduction than a water-only rinse for *S*. *aureus* and *E*. *coli*, respectively. The 2.5 ±0.5 log reduction of *E*. *coli* on VITRO-SKIN^®^ was very similar to the 2.9 ±0.7 log reduction obtained for hands washed with soap (see above).

### Scenario Simulations

The bacterial count on a human hand or on a fomite was calculated in scenarios describing different contamination routes. Calculations were made using the TR Model, applying the transfer rates shown in Table 1 for VITRO-SKIN^®^, stainless steel and laminate.

**Fig 4:**
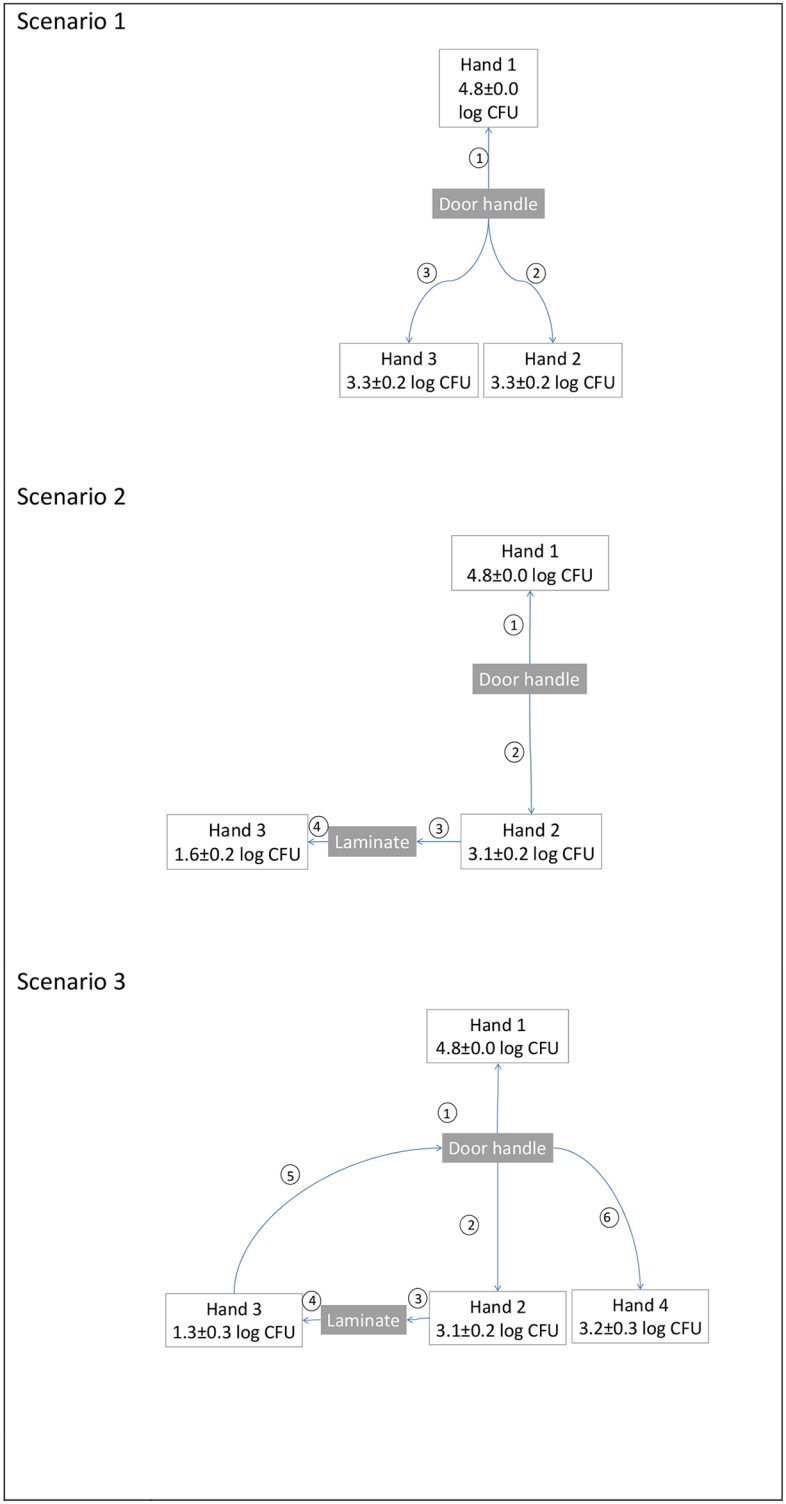
Illustrations of different contamination scenarios where bacteria are transferred between hands and surfaces. The count on Hand 1 is assumed to be 5 log CFU at the beginning of the scenario and the final count after each scenario is shown. The order of events in the scenario is indicated in the circles. The standard deviation is a result of the variation in the transfer rates for VITRO-SKIN^®^, stainless steel and laminate.

Three different scenarios were simulated to illustrate the spread of *S*. *aureus* starting from a contaminated hand (Hand 1) (Fig 4). Hand 1 was assumed to initially have 5.0 log CFU *S*. *aureus* on the fingertips while Hand 2, Hand 3 and Hand 4 were assumed to be initially uncontaminated, i.e. harbour 0 log CFU *S*. *aureus*. Hand 1, Hand 2, Hand 3 and Hand 4 were assumed to belong to different persons. The final levels on the hands are shown in Fig 4.

Scenario 1. Hand 1 touched a stainless steel door handles. The door handle was touched by Hand 2 and subsequently by Hand 3. At the end of the scenario, all the hands were contaminated with *S*. *aureus* and the level decreased in the order: Hand 1>Hand 2 = Hand 3. The contaminated door handle was able to spread a high level of *S*. *aureus* to uncontaminated hands.
Scenario 2. Hand 1 touched a door handle that was subsequently touched by Hand 2. Hand 2 touched a laminate bench top. Hand 3 touched the same spot on the bench. All hands became contaminated. The laminate was contaminated with the bacteria that Hand 2 transferred from the door handle. The *S*. *aureus* level of Hand 3 was lower than of Hand 2 since it touched laminate that was contaminated by the bacteria transferred from Hand 2.
Scenario 3. Hand 1 touched stainless steel door handle. Hand 2 touched the same door handle and then touched a laminate bench top. Hand 3 touched the bench top touched by Hand 2 and then touched the door handle. Hand 4 touched the door handle that had been touched by Hand 1 and Hand 3. The *S*. *aureus* level of Hand 3 was lower than of Hand 2 since it came later in the contamination chain. However, the level of *S*. *aureus* on Hand 4 was higher since it has been contaminated from the door handle to which the bacteria were transferred from both Hand 1 and Hand 3.

The scenario simulation shows that one contaminated hand can spread harmful bacteria to surfaces that spread the contamination further to new hands. It illustrates that it is easy for an entire environment to become infected.

### Simulation of Interventions

If interventions like hand washing and cleaning of surfaces are introduced, the amount of *S*. *aureus* on the hands will be different at the end of the above scenarios. Scenario 2 was used to study the effect of interventions (Tables 4, 5 and 6).

**Table 4:** Predicted count of *S*. *aureus* on hands in scenario 2 with and without multiple interventions.

Interventions	*S*. *aureus* transferred and remaining on hands (log CFU)		
	Hand 1	Hand 2	Hand 3
No intervention	4.8±0.0	3.1±0.2	1.6±0.2
Washing Hand 1 ^1)^	2.8±0.4	1.1±0.4	<1 CFU
Cleaning door handle ^2)^	4.8±0.0	<1 CFU	<1 CFU
Cleaning the laminate bench ^2)^	4.8±0.0	3.1±0.2	2.7±0.3 CFU
Washing Hand 1 and 2 ^1)^	2.8±0.4	<1 CFU	<1 CFU

^1)^ Washing was assumed to reduce *S*. *aureus* by 2.0±0.4 log CFU according to the experiment on VITRO-SKIN^®^

^2)^ Cleaning the door handle and laminate bench was assumed to reduce *S*. *aureus* by 3.3±0.1 log CFU and 0.5±0.7 log CFU respectively, according to the results of reduction using white cleaning wipes with Allrengöring and low humidity (Table 2).

**Table 5:** Predicted effect of hand washing on the count of *S*. *aureus* in scenario 2.

Interventions	*S*. *aureus* transferred and remaining on hands after the scenario (log CFU)		
	Hand 1	Hand 2	Hand 3
No intervention	4.8±0.0	3.1±0.2	1.6±0.2
Washing hand 1 with water rinse^1)^	4.3±0.1	2.6±0.2	1.1±0.2
Washing hand 1 with soap solution^2)^	2.8±0.4	1.1±0.4	<1 CFU
Washing hands 1 and 2 with water rinse ^1)^	4.3±0.1	2.1±0.2	1.1±0.2
Washing hands 1 and 2 with soap ^2)^	2.8±0.4	<1 CFU	<1 CFU

^1)^ Washing hands with only a water rinse was assumed to reduce *S*. *aureus* by 0.5±0.1 log CFU (according to the experiment on VITRO-SKIN^®^).

^2)^ Washing with a soap solution was assumed to reduce *S*. *aureus* by 2.0±0.4 log CFU (according to the experiment on VITRO-SKIN^®^).

**Table 6:** Predicted effect of cleaning the door handle on the count of *S*. *aureus* in scenario 2.

Interventions	*S*. *aureus* transferred and remaining on hands after the scenario (log CFU)		
	Hand 1	Hand 2	Hand 3
No intervention	4.8±0.0	3.1±0.2	1.6±0.2
Cleaning door handle (2 log reduction)	4.8±0.0	1.1±0.2	<1 CFU
Cleaning door handle (3 log reduction)	4.8±0.0	~1 CFU	<1 CFU
Cleaning door handle (5 log reduction)	4.8±0.0	<1 CFU	<1 CFU

Cleaning the door handle would result in less *S*. *aureus* on Hand 2 and Hand 3 (Table 4). Cleaning the laminate bench top would only affect Hand 3. Washing Hand 1 and Hand 2 before touching the surface would be effective in reducing the contamination. This simulation clearly shows the need for multiple interventions.

Calculations were performed to compare the use of soap and rinsing with water when washing hands, using scenario 2 as a reference of no intervention/hand washing (Table 5). The calculated number of *S*. *aureus* on Hand 2 and Hand 3 were 0.5 log CFU lower if Hand 1 was rinsed with water. The use of soap resulted in a greater reduction on all hands. Hand 2 and Hand 3 were >1 log CFU lower if Hand 1 was washed with soap instead of water only.

The effect of the cleaning varies depending on the efficiency of the cleaning system (cleaning agent, moisture level, materials). Predictions were made for cleaning, resulting in 2 log CFU, 3 log CFU or 5 log CFU reduction of *S*. *aureus* on the door handle. The calculated number of *S*. *aureus* on Hand 2 in the scenario 2 was reduced from 3.1 log CFU to 1.1 log CFU, ~1 CFU or < 1 CFU (Table 6) for various cleaning efficacies.

### Validation of Simulations

**Fig 5:**
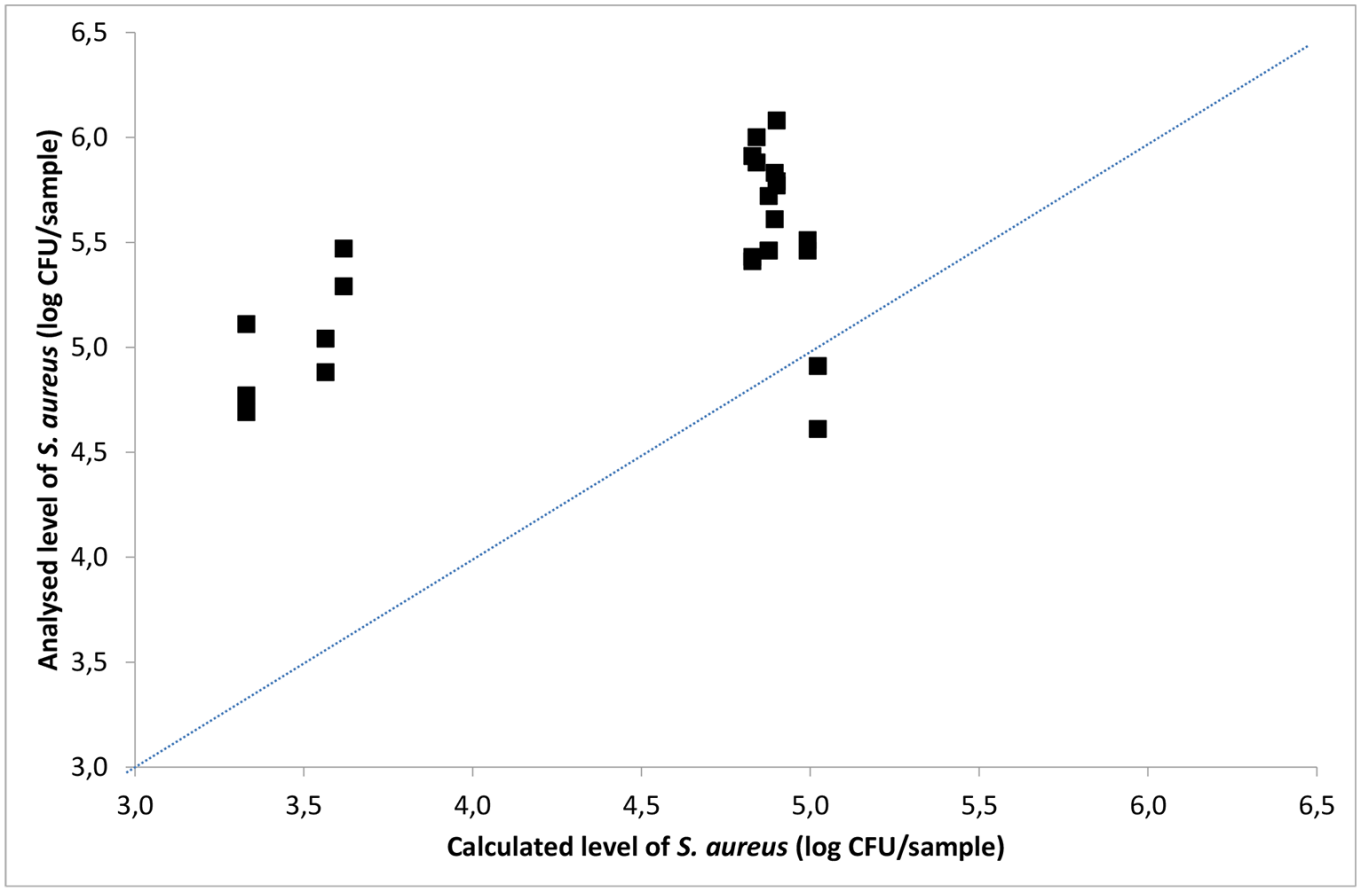
Analysed versus calculated level of *S*. *aureus* on VITRO-SKIN^®^ after simulating three contamination scenarios. A line of equivalence is shown.

The analysed bacterial levels from experiments performed by blotting VITRO-SKIN^®^ and stainless steel surfaces, applying scenarios 1–3, were used to validate the TR model. In the scenarios, stainless steel was used as the surface material. Comparison was made between the number of *S*. *aureus* analysed on the VITRO-SKIN^®^ and the number calculated using the TR model (Fig 5). A linear relationship was obtained between the analysed level of *S*. *aureus* and the calculated level, if assuming two of the results being out layers.

The calculated level of *S*. *aureus* on the VITRO-SKIN^®^ in the scenarios was generally lower than the analysed level. The only exception was the VITRO-SKIN^®^ simulation Hand 3 in a variant of scenario 3, where hand 3 did not touch the door handle. The lower calculated values indicates that at least one of the transfer rates evaluated from blotting series (VITRO-SKIN^®^ to stainless steel or stainless steel to VITRO-SKIN^®^) used for the calculation was lower than the actual transfer rates in the validation scenario, due to differences in the experimental set-ups when developing the model as opposed to when validating the model.

## Discussion

The TR model that was developed offers flexible ways to improve hygiene management. It may be used for the evaluation of contamination scenarios, including fomites and human hands, and for the assessment and identification of possible and suitable intervention steps to reduce bacterial levels. The model makes it possible to predict the effect of different cleaning routines and hand washing routines on cross contamination. Finally, it enables the prediction of how different materials affect the extent of contamination. This is important when choosing appropriate materials that do not facilitate microbial contamination on surfaces of e.g. bench top, furniture or equipment. Not only can the simulation model be used to improve hygiene management, it is a useful tool for training personal in proper hygiene practices.

Quantitative modelling of contamination scenarios requires reliable data on transfer rates, relevant to the specific bacteria and type of surfaces under evaluation [10]. The better the transfer data, the more precise is the information on the extent of contamination. Depending on the experimental set-up used to determine the transfer rates, there will be ranges of standard deviations and uncertainties in the ability to control parameters affecting the known and unknown transfer rates. Key factors affecting the transfer rate include moisture, pressure, time and inoculum size [11] [23]. Most data in the literature for transfer rates on contamination involving human hands/fingers are based on a two-point determination of bacterial levels; before and after touching a surface [16] [24] [25]. The use of fingers and two-point determination could lead to a wide spread of results of transfer rates because of the difficulty in controlling the key factors. This is exemplified by the transfer rates of *E*. *aerogenes* between spigots and clean hands, which are reported to vary in 30 replicates between 0.021% and 72.4% [24].

The present study used an experimental set-up, using VITRO-SKIN^®^ with an improved possibility to control of moisture, temperature and pressure, in an attempt to reduce the large variation in transfer rates. Furthermore, the transfer rates were calculated from series of blotting (n = 24). This enabled a more accurate determination of the transfer rate, calculated as the slope of several determinations of bacterial levels. Blotting series have been used when determining transfer of bacteria from surfaces in other situations, such as potato tissue [26] and dental implant [27]. Using VITRO-SKIN^®^ in the present study, the transfer rate of *S*. *aureus* showed low standard deviations. The standard deviation in transfer rates was between 0.2%-2.7% for five surface materials in this study. However, the transfer rate for textile showed a high standard deviation.

It would be easier to conduct studies on pathogenic bacteria if experiments did not involve fingers/hands of volunteers. Due to safety concerns, hand washing products and hand washing disinfectants are evaluated in compliance with the European EN 1500:1997 standard test, using a non-pathogenic strain of *E*. *coli*. A lot of information is available about this specific strain, but information about several other pathogenic bacteria is also needed. To our knowledge, the present study is the first to use artificial skin for determination of transfer rates of pathogenic bacteria and their reduction during hand washing. Desai et al (2011) [15] used an imprinting technique to evaluate the transfer between fomites and pig skin. The artificial skin VITRO-SKIN^®^ is used for evaluation of cosmetic formulas [28]. VITRO-SKIN^®^ is developed to simulate the properties of human skin in respect of topography, pH, critical surface tension and ionic strength. In the present study, a test strain of *E*. *coli* during washing of VITRO-SKIN^®^ was reduced by the same amount as with the standard method using test persons.

In the present study, the transfer rate of *E*. *coli* from fingers to stainless steel, measured using the two-point method, was higher (~75%) than the transfer rate (~46%) obtained from blotting series of VITRO-SKIN^®^ to stainless steel. Calculating transfer rates, 75% may not in fact be different than 46%. Due to different methodologies different numbers of replicates are used. Ten replicates were used for the two-point method using test persons and three replicate blotting series including each 21 blottings for the method using VITRO-SKIN^®^.

The experimental set-up should resemble real conditions as far as possible. More work is needed to close the gap between lab conditions using human hands/fingers or artificial skin, and the conditions in settings such as hospital wards. Humidy is one important factor influencing transfer rates according to Pérez-Rodrígues et al. (2008) [11]. The possibility to control the humidity level using artificial skins is an advantage as opposed to human hands.

For hygiene management purposes, it is crucial to understand the effect of different cleaning strategies. The extent to which cleaning reduces bacteria on a surface is affected by several factors. These include the cleaning agent, surface material, soiling and humidity as reported in the present study and in other studies [17]. It is important to evaluate and take into consideration the variation in the cleaning effect, due to cleaning parameters, when studying cross contamination scenarios [9]. In a review, Pittet et al (2006) [29] conclude that many mathematical models simulating the effect of compliance to hand hygiene assume that 100% of the specific bacteria is removed when hands are cleaned, and the models do not take into account the reduction level depending on the hand washing efficiency. The efficiency of both hand washing and surface cleaning should be included when intervention strategies are designed.

## Conclusion

Quantitative modelling of contamination scenarios by calculating the bacterial transfer between hands and surfaces, including intervention strategies like hand washing and cleaning of surfaces, provides knowledge and data on which to base decisions about hygiene management. A model is a simplification of a real situation and contamination routes are complex scenarios. However, the results are largely dependent on the input data and on how accurate they are for the question raised and the actual situation being simulated. How to perform the correct test to achieve the correct data is not a straightforward matter. There are advantages to producing data on transfer rates and reductions in a laboratory using artificial skin like VITRO-SKIN^®^ because it makes it possible to control different parameters to evaluate the specific effect. However, the data needs to be relevant to real conditions. Further development of methodology using artificial systems that simulate real-life situations would make it easier to obtain more data to improve hygiene assessment and management.

## Supporting Information

S1 File Data Table 1. (PDF)

S2 File Data Table 2. (PDF)

S3 File Data_Transfer E coli ss-Vitro Skin-human skin. (PDF)

S4 File Data_Washing hands contaminated with Ecoli. (PDF)

S5 File Data_Washing Vitro Skin. (PDF)
