# Supplementary material for: Transfer and Decontamination of S. aureus in Transmission Routes Regarding Hands and Contact Surfaces
Source: PLoS One. 2016 Jun 9;11(6):e0156390. doi: 10.1371/journal.pone.0156390 (PMC4900614; doi:10.1371/journal.pone.0156390)
Supplement: S1 File — (PDF) [file pone.0156390.s001.pdf]

Table 1 Raw data for calculated transfer rate of S. aureus

From contaminated	To material	Experimen	Blotting nc	log CFU/su	Experimen	Blotting nc	log CFU/su
VITRO SKIN	Plastic apron	1	1	5,84	2	1	6,01
VITRO SKIN	Plastic apron	1	3	5,63	2	3	5,57
VITRO SKIN	Plastic apron	1	6	5,37	2	6	4,78
VITRO SKIN	Plastic apron	1	9	4,82	2	9	4,32
VITRO SKIN	Plastic apron	1	12	4,06	2	12	3,89
VITRO SKIN	Plastic apron	1	15	3,73	2	15	3,00
VITRO SKIN	Plastic apron	1	18	3,24	2	18	2,45
VITRO SKIN	Plastic apron	1	21	2,97	2	21	2,16
VITRO SKIN	Cardboard	1	1	6,23	2	1	6,28
VITRO SKIN	Cardboard	1	3	5,80	2	3	6,09
VITRO SKIN	Cardboard	1	6	4,88	2	6	5,37
VITRO SKIN	Cardboard	1	9	4,47	2	9	4,65
VITRO SKIN	Cardboard	1	12	3,32	2	12	4,02
VITRO SKIN	Cardboard	1	15	3,19	2	15	3,40
VITRO SKIN	Cardboard	1	18	3,22	2	18	3,19
VITRO SKIN	Cardboard	1	21	2,75	2	21	NA
VITRO SKIN	Stainless Steel	1	1	6,32	2	1	6,37
VITRO SKIN	Stainless Steel	1	3	5,84	2	3	5,80
VITRO SKIN	Stainless Steel	1	6	5,16	2	6	5,09
VITRO SKIN	Stainless Steel	1	9	4,62	2	9	4,32
VITRO SKIN	Stainless Steel	1	12	4,94	2	12	3,56
VITRO SKIN	Stainless Steel	1	15	2,80	2	15	3,42
VITRO SKIN	Stainless Steel	1	18	2,08	2	18	3,07
VITRO SKIN	Stainless Steel	1	21	NA	2	21	2,49
VITRO SKIN	HDPE	1	1	5,89	2	1	5,72
VITRO SKIN	HDPE	1	3	6,49	2	3	5,42
VITRO SKIN	HDPE	1	6	5,01	2	6	4,78
VITRO SKIN	HDPE	1	9	3,56	2	9	4,18
VITRO SKIN	HDPE	1	12	3,86	2	12	3,78
VITRO SKIN	HDPE	1	15	3,21	2	15	3,38
VITRO SKIN	HDPE	1	18	3,39	2	18	2,35
VITRO SKIN	HDPE	1	21	2,47	2	21	2,06
VITRO SKIN	Laminate	1	1	6,12	2	1	6,00
VITRO SKIN	Laminate	1	3	5,43	2	3	5,43
VITRO SKIN	Laminate	1	6	4,78	2	6	4,18
VITRO SKIN	Laminate	1	9	3,91	2	9	3,53
VITRO SKIN	Laminate	1	12	3,19	2	12	3,23
VITRO SKIN	Laminate	1	15	2,53	2	15	2,45
VITRO SKIN	Laminate	1	18	2,16	2	18	2,53
VITRO SKIN	Laminate	1	21	2,27	2	21	1,43
VITRO SKIN	Textile	1	1	6,34	2	1	6,51
VITRO SKIN	Textile	1	3	4,20	2	3	3,76
VITRO SKIN	Textile	1	6	3,68	2	6	3,45
VITRO SKIN	Textile	1	9	3,76	2	9	3,45
VITRO SKIN	Textile	1	12	3,71	2	12	3,43
VITRO SKIN	Textile	1	15	3,57	2	15	3,28
VITRO SKIN	Textile	1	18	3,50	2	18	3,02
VITRO SKIN	Textile	1	21	3,22	2	21	2,75

VITRO SKIN	Tile	1	1	6,17	2	1	6,25
VITRO SKIN	Tile	1	3	5,94	2	3	5,82
VITRO SKIN	Tile	1	6	4,92	2	6	4,82
VITRO SKIN	Tile	1	9	4,45	2	9	4,31
VITRO SKIN	Tile	1	12	4,09	2	12	3,76
VITRO SKIN	Tile	1	15	3,36	2	15	3,23
VITRO SKIN	Tile	1	18	3,02	2	18	2,86
VITRO SKIN	Tile	1	21	3,16	2	21	2,26

Raw data for calculated transfer of E. coli

VITRO SKIN	Stainless steel	1	1	7,28	2	1	7,40
		1	3	6,26	2	3	6,48
		1	6	3,95	2	6	5,12
		1	9	########	2	9	4,73
		1	12	2,48	2	12	3,88
		1	15	1,48	2	15	3,04
		1	18	1,26	2	18	3,23
		1	21	1,08	2	21	3,34

Experimen	Blotting nc	log CFU/surface
3	1	5,81
3	3	5,42
3	6	5,92
3	9	4,40
3	12	3,80
3	15	3,00
3	18	2,87
3	21	2,37
3	1	6,32
3	3	5,65
3	6	5,02
3	9	4,19
3	12	3,26
3	15	2,93
3	18	2,45
3	21	2,75
3	1	6,18
3	3	5,82
3	6	5,01
3	9	4,29
3	12	3,06
3	15	2,89
3	18	2,33
3	21	1,91
3	1	5,73
3	3	5,68
3	6	5,03
3	9	4,13
3	12	3,55
3	15	3,02
3	18	3,32
3	21	2,92
3	1	5,89
3	3	5,53
3	6	4,85
3	9	3,52
3	12	2,95
3	15	2,35
3	18	2,61
3	21	1,80
3	1	6,18
3	3	4,20
3	6	3,52
3	9	3,34
3	12	3,42
3	15	3,23
3	18	3,28
3	21	2,98

3	1	5,95
3	3	5,57
3	6	4,89
3	9	4,26
3	12	3,76
3	15	2,92
3	18	2,32
3	21	2,33

3	1	7,48
3	3	7,02
3	6	5,16
3	9	4,63
3	12	4,11
3	15	3,26
3	18	2,48
3	21	1,92
