# Supplementary material for: Transfer and Decontamination of S. aureus in Transmission Routes Regarding Hands and Contact Surfaces
Source: PLoS One. 2016 Jun 9;11(6):e0156390. doi: 10.1371/journal.pone.0156390 (PMC4900614; doi:10.1371/journal.pone.0156390)
Supplement: S2 File — (PDF) [file pone.0156390.s002.pdf]

**Cleaner**	**Wipe**	**Humidity**			
					**Before** [illegible]
			Stainless steel+ BA	Stainless steel+Egg	Laminat+BA
Allrengöring	Blue	High	6,53; 6,59; 6,88	7,29; 7,26; 7,21	6,66; 6,55; 6,63
	Blue	Low	6,53; 6,59; 6,88	7,29; 7,26; 7,21	6,66; 6,55; 6,63
	White	High	6,84; 6,88; 6,84	7,2; 7,3; 8,13	6,66; 6,55; 6,63
	White	Low	6,84; 6,88; 6,84	7,2; 7,3; 8,13	6,66; 6,55; 6,63
Virkon	Blue	High	3,83; 4,0; 4,1	7,36; 7,07; 7,16	6,71; 6,43; 6,73
	Blue	Low	3,83; 4,0; 4,1	7,36; 7,07; 7,16	6,71; 6,43; 6,73
	White	High	3,83; 4,0; 4,1	7,36; 7,07; 7,16	6,71; 6,43; 6,73
	White	Low	3,83; 4,0; 4,1	7,36; 7,07; 7,16	6,71; 6,43; 6,73
Dags 70	Blue	High	5,71; 6,38; 5,8	7,13; 7,1; 7,08	6,8; 6,71; 6,48
	Blue	Low	5,71; 6,38; 5,8	7,13; 7,1; 7,08	6,8; 6,71; 6,48
	White	High	5,71; 6,38; 5,8	7,13; 7,1; 7,08	6,8; 6,71; 6,48
	White	Low	5,71; 6,38; 5,8	7,13; 7,1; 7,08	6,8; 6,71; 6,48

				Level S. aureus (log CF
:leaning				
Laminate+Egg	Tile+BA	Tile+Egg	Stainless steel+ BA	Stainless steel+Egg
7,24; 7,14; 7,29	6,80; 6,73; 6,86	7,26; 7,16; 7,18	<0,48; <0,48; <0,48	1,38; 1,8; 1,38
7,24; 7,14; 7,29	6,80; 6,73; 6,86	7,26; 7,16; 7,18	<0,48; 1,26; 2,94	2,38; 2,88; 3,71
7,24; 7,14; 7,29	6,80; 6,73; 6,86	7,26; 7,16; 7,18	1,78; 1,38; 1,82	1,59; <0,4;, <0,48
7,24; 7,14; 7,29	6,80; 6,73; 6,86	7,26; 7,16; 7,18	3,59; 3,49; 3,62	1,67; 2,55; 1,89
7,18; 7,34, 7,11	6,65; 6,78; 6,65	6,08; 6,95, 6,10	<0,48; 1,59; 1,59	<0,48; 0,48; <0,48
7,18; 7,34, 7,11	6,65; 6,78; 6,65	6,08; 6,95, 6,10	<0,48, 1,78; 1,32	4,59; 4,14; 6,84
7,18; 7,34, 7,11	6,65; 6,78; 6,62	6,8; 6,95; 7,1	<0,48; <0,48; 0,79	0,78; 1,38; 1,08
7,18; 7,34, 7,11	6,65; 6,78; 6,62	6,8; 6,95; 7,1	0,48; <0,48; 1,98	2,05, 2,18; 3,07
7,03; 7,28; 7,12	5,59; 6,22; 6,5	7,08; 7,28; 7,18	0,78; 1,18; 0,48	4,03; 3,73; 5,73
7,03; 7,28; 7,12	5,59; 6,22; 6,5	7,08; 7,28; 7,18	3,84; 3,33; 2,37	6,66; 7,16; 6,53
7,03; 7,28; 7,12	5,59; 6,22; 6,5	7,08; 7,28; 7,18	1,48; 1,08; <0,48	4,49; 2,36; 2,06
7,03; 7,28; 7,12	5,59; 6,22; 6,5	7,08; 7,28; 7,18	3,68; 2,02; 2,03	6,13; 6,36; 6,69

-U/surface)

After cleaning			
Laminat+BA	Laminate+Egg	Tile+BA	Tile+Egg
2,55; 2,73; 2,69	4,07; 3,9; 3,24	0,48; 0,48; 0,48	2,91; 0,48; 0,48
3,77; 2,36; 3,17	6,11; 5,38; 7,03	0,95; 0,48; 0,95	6,89; 2,8; 6,34
2,94; 3,11; 3,53	3,43; 4,8; 4,2	2,49; 0,48; 2,93	2,54; 1,82; 2,39
4,6; 4,6; 4,97	6,63; 6,86; 6,62	3,5; 3,3; 3,06	6,95; 4,17; 4,71
1,08; 1,59; 1,18	3,18; 3,91, 3,65	0,78; 1,8; 1,95	1,26; 2,22; 0,48
1,08; 0,48; 3,41	6,45; 4,18; 4,76	1,08; 1,92; 1,18	6,18; 2,63; 5,72
1,18; 2,95; 1,18	2,82; 1,68; 2,03	0,48; 1,08; 0,78	2,44; 0,95; 2,95
3,89; 3,25; 3,22	6,76; 6,97; 6,44	1,98; 5,04; 1,94	5,59; 4,83; 3,33
2,12; 1,91; 3,05	5,73; 4,85; 5,2	0,48; 3,94; 1,86	5,34; 4,67; 5,26
4,08; 3,8; 4,6	6,76; 6,61; 7,12	5,06; 3,62; 1,43	6,97; 7,18; 6,96
<0,48; 2,83; 1,0[illegible]	5,8; 5,39; 5,98	3,97; 2,33; 2,28	4,39; 1,08; 2,61
6,01; 5,32; 4,71	6,66; 6,38; 6,71	2,49; 3,56; 4,56	6,37; 5,84; 5,35
