# Supplementary material for: Transfer and Decontamination of S. aureus in Transmission Routes Regarding Hands and Contact Surfaces
Source: PLoS One. 2016 Jun 9;11(6):e0156390. doi: 10.1371/journal.pone.0156390 (PMC4900614; doi:10.1371/journal.pone.0156390)
Supplement: S3 File — (PDF) [file pone.0156390.s003.pdf]

Transfer E. coli between stainless steel and human skin

Antal prov	Infected fingers against clean surface			Clean fingers a[illegible]
	log prevalue	log postvalue	log reduktionsfaktor	log prevalue, yt[illegible]
1	5,33	2,70	2,63	5,76
2	6,31	5,11	1,20	5,38
3	5,68	4,56	1,13	5,68
4	5,66	4,51	1,15	5,86
5	5,43	5,79	-0,36	5,97
6	3,40	5,75	-2,35	5,76
7	6,38	5,54	0,84	5,38
8	2,00	2,40	-0,40	5,68
9	5,04	4,99	0,05	5,86
10	5,13	3,02	2,11	5,97
**mean**	**5,04**	**4,44**	**0,60**	**5,73**
**s**	**1,35**	**1,28**	**1,43**	**0,21**
**n**	**10**	**10**	**10**	**10**

		Infected fingers
	Reducktion (%)	75

Transfer E.coli between stainless steel and VITRO SKIN

From contaminatec	To material	Experiment	Blotting no	log CFU/surface
VITRO SKIN	Stainless steel	1	1	7,28
VITRO SKIN	Stainless steel	1	3	6,26
VITRO SKIN	Stainless steel	1	6	3,95
VITRO SKIN	Stainless steel	1	9	NA
VITRO SKIN	Stainless steel	1	12	2,48
VITRO SKIN	Stainless steel	1	15	1,48
VITRO SKIN	Stainless steel	1	18	1,26
VITRO SKIN	Stainless steel	1	21	1,08
Stainless steel	VITRO SKIN	1	1	NA
Stainless steel	VITRO SKIN	1	3	NA
Stainless steel	VITRO SKIN	1	6	NA
Stainless steel	VITRO SKIN	1	9	2,30
Stainless steel	VITRO SKIN	1	12	2,00
Stainless steel	VITRO SKIN	1	15	2,30
Stainless steel	VITRO SKIN	1	18	2,00
Stainless steel	VITRO SKIN	1	21	NA

gainst contaminated surface

log postvalue, yt	log reduktionsfaktor
3,63	2,13
5,25	0,13
4,92	0,76
4,64	1,21
4,68	1,29
4,56	1,20
4,61	0,77
5,69	-0,01
5,62	0,24
5,34	0,63
**4,89**	**0,84**
**0,61**	**0,65**
**10**	**10**

	Infected surface
Reduction (%)	85

**Experiment**	**Blotting no**	**log CFU/su**	**Experimen**	**Blotting nc**	**log CFU/surface**
2	1	7,40	3	1	7,48
2	3	6,48	3	3	7,02
2	6	5,12	3	6	5,16
2	9	4,73	3	9	4,63
2	12	3,88	3	12	4,11
2	15	3,04	3	15	3,26
2	18	3,23	3	18	2,48
2	21	3,34	3	21	1,92
2	1	2,98	3	1	4,20
2	3	4,99	3	3	5,47
2	6	4,49	3	6	5,07
2	9	4,64	3	9	4,62
2	12	3,73	3	12	4,20
2	15	3,26	3	15	4,49
2	18	4,69	3	18	4,77
2	21	4,51	3	21	4,38
