# Supplementary material for: Transfer and Decontamination of S. aureus in Transmission Routes Regarding Hands and Contact Surfaces
Source: PLoS One. 2016 Jun 9;11(6):e0156390. doi: 10.1371/journal.pone.0156390 (PMC4900614; doi:10.1371/journal.pone.0156390)
Supplement: S4 File — (PDF) [file pone.0156390.s004.pdf]

Sample

Sample	Soap			
	log prevalue	log postvalue	log reduktionsfaktor	log prevalue
1	6,15	2,28	3,87	6,58
2	5,71	1,87	3,84	5,38
3	5,96	2,65	3,31	6,72
4	5,49	2,43	3,06	6,08
5	6,04	3,29	2,74	5,94
6	6,10	2,80	3,31	4,78
7	5,88	3,61	2,27	5,99
8	4,40	2,76	1,64	5,86
9	5,58	3,29	2,29	5,96
10	6,03	2,63	3,40	5,91
11	5,48	3,00	2,48	5,53
12	5,28	2,83	2,45	6,17
Mean	5,71	2,78	2,93	5,91
sd	0,50	0,50	0,70	0,52
n	12	12	12	12

Time	Wash with soap solution		Wash with soap solution and subsequent deci	
Before was	5,71	0,50	5,91	0,52
After wash	2,78	0,50	1,77	1,00

Soap + disinfection

log postvalue	log reduktionsfaktor
1,88	4,70
1,15	4,23
2,91	3,81
2,76	3,31
1,91	4,03
0,30	4,48
1,72	4,28
2,79	3,07
0,85	5,12
0,00	5,91
2,59	2,94
2,36	3,81
1,77	4,14
1,00	0,86
12	12

nfection
