# Supplementary material for: Transfer and Decontamination of S. aureus in Transmission Routes Regarding Hands and Contact Surfaces
Source: PLoS One. 2016 Jun 9;11(6):e0156390. doi: 10.1371/journal.pone.0156390 (PMC4900614; doi:10.1371/journal.pone.0156390)
Supplement: S5 File — (PDF) [file pone.0156390.s005.pdf]

Replicate	Prov	S.aureus	E.coli
1	Inoculated skin	7,55; 7,58; 7,56	7,78; 7,90; 7,84
1	Inoculated skin washed with water	7,23; 7,17; 7,20	6,38; 6,43; 6,41
1	Inoculated skin washed with soap and water	5,47; 5,73; 5,49	5,41; 5,68; 5,51
2	Inoculated skin	7,48; 7,52; 7,50	7,73; 7,62; 7,68
2	Inoculated skin washed with water	6,84; 6,95; 6,89	6,18; 6,49; 6,33
2	Inoculated skin washed with soap and water	6,05; 5,61; 5,48	5,66; 5,45; 5,87
3	Inoculated skin	7,57; 7,67; 7,62	7,54; 7,77; 7,66
3	Inoculated skin washed with water	6,96; 7,09; 7,02	5,40; 5,72; 5,56
3	Inoculated skin washed with soap and water	5,52; 5,67; 4,78	4,68; 4,49; 4,59
